# Intra- and interspecific competition resulting from spatial coexistence among larvae of closely-related caddisflies from the genus *Hydropsyche*

**DOI:** 10.7717/peerj.13576

**Published:** 2022-06-23

**Authors:** Mariusz Tszydel, Dagmara Błońska

**Affiliations:** Faculty of Biology and Environmental Protection, Department of Ecology and Vertebrate Zoology, University of Łódź, Łódź, Poland

**Keywords:** Hydropsychidae, Trichopteran larvae, Space competition, One-on-one combats, Size of mandibles

## Abstract

Caddisfly larvae commonly inhabit freshwater ecosystems, where they often create multi-species aggregations. However, while several strategies have been developed to avoid or reduce inter- and intraspecific interactions, most species choose the same time to seek a suitable place for pupation, which can increase competition. The current study assesses the competitive interactions among larvae (5^th^ instar) of three co-existing *Hydropsych*e species, *viz. H. contubernalis*, *H. pellucidula*, and *H. modesta*, analysing their direct one-on-one interaction and various morphological features, such as size, weight, and mandibles. More than half of the interspecific conflicts ended with a draw, and 80% of intraspecific interactions with a decisive outcome. In fights between species, *H. pellucidula* was the most successful, and *H. modesta* the weakest. Our results confirm that among the larvae, competitive interactions were usually decided by body size, especially that of the head capsule. Although wider head capsule and higher weight were advantageous for ~60% of winning larvae, there were no distinct winning species. The chewing mouthpart turned out to be supportive in the fight: regardless of the species, longer and wider mandibles were significant for winning specimens, but not the distance between mandibles. Hence, acquiring a suitable place for pupation is determined by the possession of certain features enhancing the fighting potential of individual larvae, which does not exclude any species from the possibility of closing the life cycle. Future studies on interactions among caddisfly larvae could include experience in fights, volitional features and stridulation (not tested).

## Introduction

The caddisflies of the Hydropsychidae are rheophilic organisms inhabiting all types of running waters, including the gravel coastal zones of lakes on occasion. They play a significant role in the reduction of fine organic matter, especially below flow-through lakes and dam reservoirs (Georgian & Thorp, 1992; Poepperl, 2000), as well as in creating stable ground for the settlement of other groups of invertebrates (Cardinale, Gelmann & Palmer, 2004; Tumolo et al., 2019). Due to their wide range of tolerance for various pollutants, they are often used as bioindicators (Tszydel et al., 2015).

Hydropsychidae larvae can form multispecies conglomerates comprising various related species inhabiting the same habitats (Muotka, 1990; Harding, 1997; Tanida, 2007). The factors determining their distribution can be divided according to trophic or habitat preferences. For the former, net localization, mesh size, the size of the particles consumed, and the nutritional quality of the seston have strong influences on food collection (Alstad, 1978; Georgian & Wallace, 1981; Tszydel & Grzybkowska, 2011). Regarding the latter, the case of microhabitats, important roles are played by water velocity and small annual fluctuations in water flow (Edington, 1968; Philipson, 1969), temperature (Fuller & Mackay, 1980) and oxygen level (Philipson & Moorhouse, 1974; van der Geest, 2007), as well as by the grain size of the inorganic substrate used to construct the larval and pupal shelter (Statzner, Mérigoux & Leichtfried, 2005). It has also been suggested that the availability of the resources needed to construct a capture net plays a more significant role than the amount of available food and its quality (Oswood, 1979).

*Hydropsyche* spp. larvae can reach high densities under suitable conditions (ca. 10,000 individuals m^−2^; Lepneva, 1971), with closely-related *Hydropsyche* species often choosing to share similar habitats. To allow them to coexist, the species often demonstrate net modifications allowing the collection of different-sized food particles (Malas & Wallace, 1977; Petersen, 1989), diversified distributions into microhabitats (Hildrew & Edington, 1979; Cudney & Wallace, 1980) or shifts in subsequent larval stages (Mackay, 1979; Komzák, 2001). Larvae can experience spatial and temporal segregation by the use of homodynamic life cycles, *i.e.*, without a diapause, and asynchronic life cycles (Williams & Hynes, 1973; Edington & Hildrew, 1995); these larvae undergo successive moultings (*i.e.*, growth) and change their location across the same stone (Williams & Hynes, 1973; Hildrew & Edington, 1979; Sieglstetter, Agasse & Caquet, 1997). However, due to limited resources (space and food), multispecies groupings of Hydropsychidae are usually described as strongly competitive for space (Malas & Wallace, 1977; Gatley, 1988), with acts of aggression, direct fights, and even cannibalism being observed, especially on young larvae (Tanida, 1984; Dudgeon, 1997; Fairchild & Holomuzki, 2002). In addition to other Hydropsychidae, the larvae also need to compete with other aquatic invertebrates, *e.g.*, Simuliidae (Hemphill, 1988), Gammaridae (Haden et al., 1999), Plecoptera and Megaloptera (Michael & Culver, 1987). However, the main factor affecting microdistribution among the Hydropsychidae remains unclear (Thorp, 1984; Miller, 1984).

**Figure 1:**
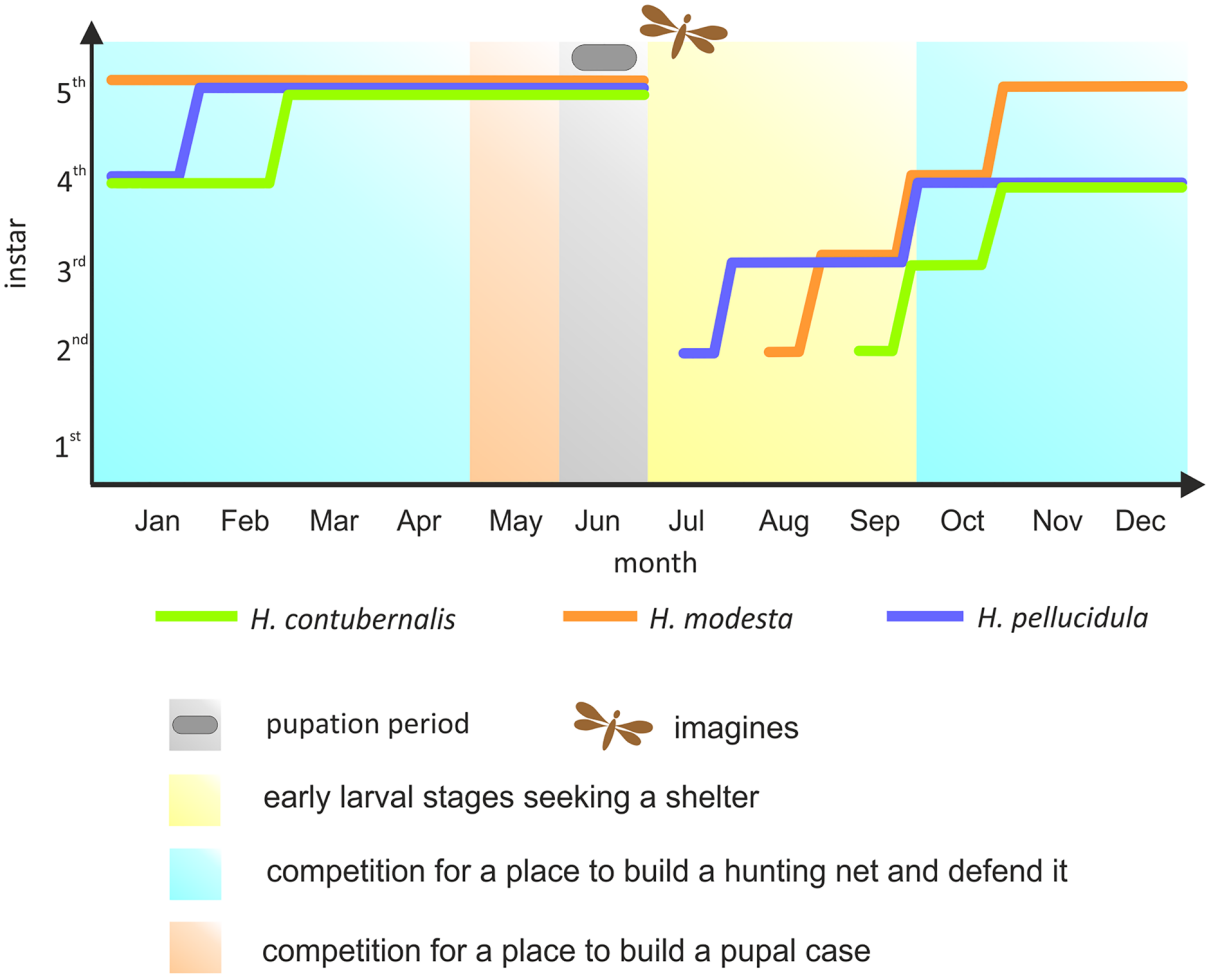
Development of the larvae of the studied species in the annual life cycle. The colored lines indicate the dominant larval stage of a particular species (based on many years of research in the river section, where individuals were obtained for the experiment). Periods of competition for various resources were marked with different colours. Pupation period and time of imago emerge were also included.

Most research has been focused on the competition among the Hydropsychidae for space to build a hunting net and shelter, as well as defending both constructions against intruders, so far. However, suitable conditions for pupation are equally important for the larvae (Statzner, Mérigoux & Leichtfried, 2005). If necessary, larvae move from their foraging location to the place where pupae are built, competing for space again. Although the larvae can avoid competition by exploiting the asynchronism between particular larval stages, pupation is nevertheless forced by the changes in water temperature and oxygen level occurring during late spring, and this life stage cannot be moved (Hildrew, 1978; Hildrew & Edington, 1979) (Fig. 1).

The aim of our study was to assess the aggression among three species of the genus *Hydropsyche* in their most advanced larval instar before pupation, which ends the larval stage. To achieve this goal, individual larvae were forced to compete for limited space; the results of these challenges were recorded and correlated with selected physical characteristics of the individuals which could have influenced the result.

## Materials and Methods

Hydropsychid larvae were collected in mid-May 2016 from the Drzewiczka River (51°27′08″N i 20°29′14″E), 2 km below the Drzewickie Reservoir dam. Among the coexisting species at this sampling site, the most common were *Hydropsyche contubernalis*, *H. pellucidula* and *H. modesta* (Tszydel, Grzybkowska & Kruk, 2009). Specimens were collected by tweezers from stones in the riffle of the main river bed. Only the 5^th^ instar, *i.e.*, the most advanced stage, was collected; all individuals were transported in aerated tanks to the laboratory. This life stage is the easiest to identify to species level due to the presence of diverse ornaments on the head capsule (frontoclypeus) and sclerotized plates (prosternites) on the ventral side of the first segment of the thorax (pronotum) (Edington & Hildrew, 1995; Neu & Tobias, 2004).

In the laboratory, each larva was kept separately in a plastic 500 ml tank. The water temperature and oxygen level was maintained at a similar level to the sampling site. The larvae were left for a 72-h acclimatization period, during which time they were not fed and allowed to empty their digestive tracts (Luoma, 1989). Food deprivation additionally increases aggression (Matczak & Mackay, 1990).

**Figure 2:**
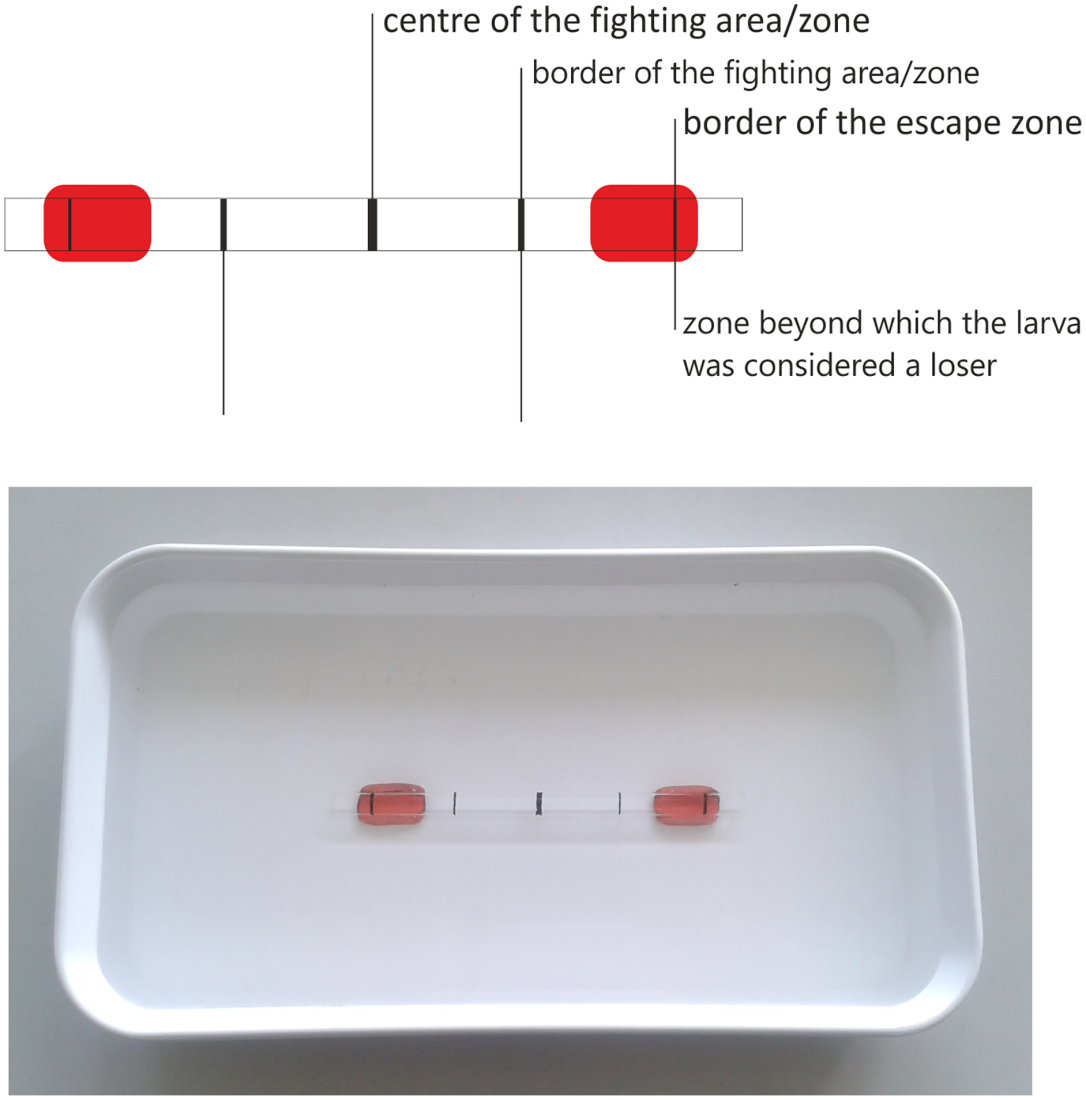
Fighting arena used in the experiment with zones enabling the result assessment.

The experimental arena consisted of a water-filled glass pipe (6 mm diameter) attached to the plastic cuvette (Fig. 2). This “combat arena” was intended to imitate the crevices available under natural conditions, which are used to find a shelter and build a pupal case. The pipe diameter enabled the larva to move ahead freely, but not to turn rapidly or reverse direction. In this situation, the larva could only move forward and attack, while retreating was hindered. It was assumed that the larva would retreat only if it realizes that it has no chance in a confrontation with its opponent.

**Figure 3:**
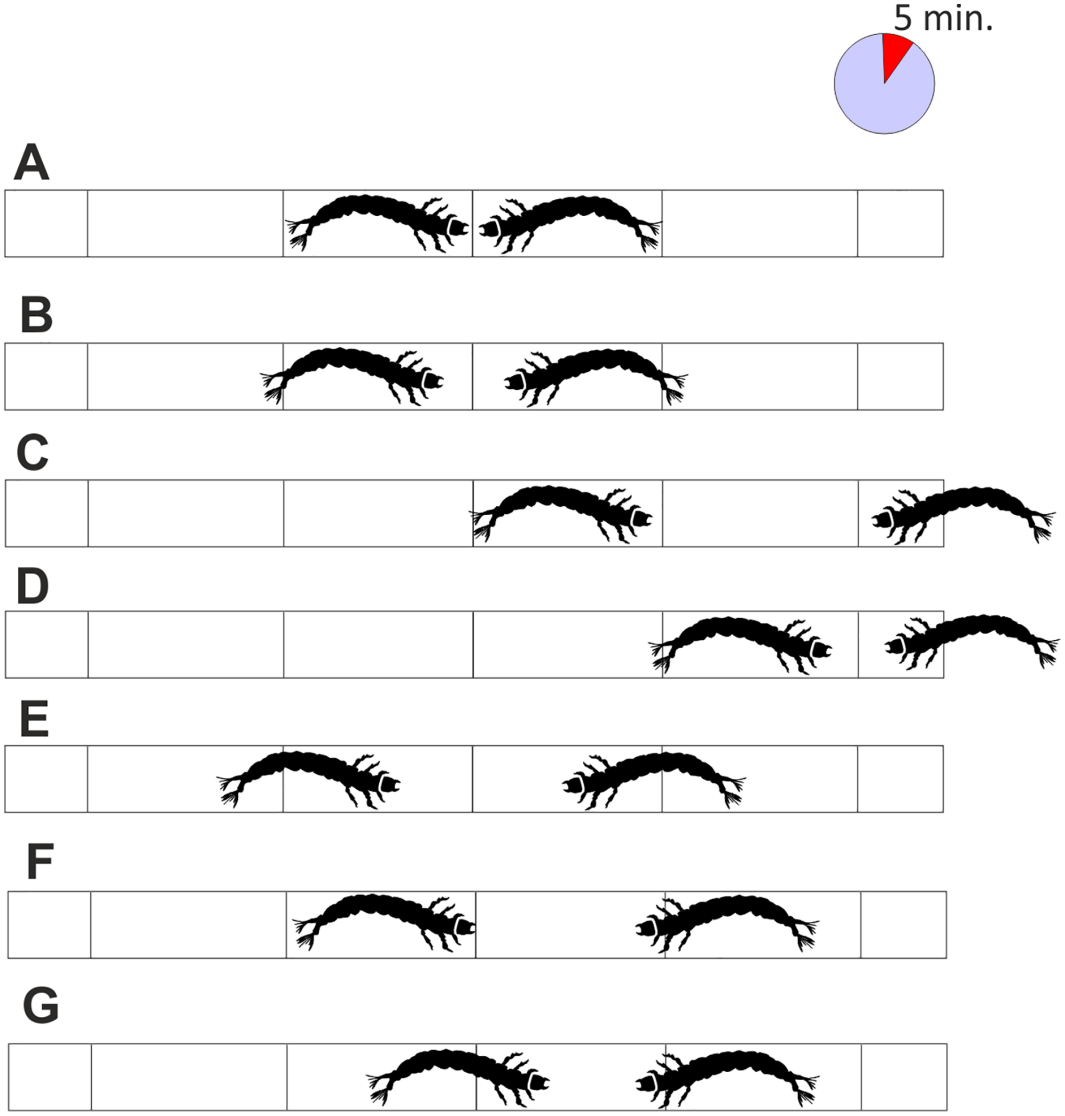
Interactions between tested larvae with the interpretation of the results. (A) the initial situation, (B) the temporary waiting, (C) and (D) the victory of one of the larvae, (E–G) draw due to retreat of both larvae, retreat of one of the larvae or lack of advantage of particular larvae. Each fight lasted 5 min.

As the pipe was transparent, it was possible to observe the conflicts and record the result: a victory was indicated when one larva passed the attack space line and the other retreated (Figs. 3C and 3D), a defeat was scored when the larva passed the escape zone, and a draw when the larvae did not fight at all, or the results were ambiguous, or neither larva gained any advantage (Figs. 3E–3G). Based on the published results (Jansson & Vuoristo, 1979; Englund & Olsson, 1990) and those of our preliminary study, the length of each battle was set to 5 min. The larvae were selected randomly, and each larva was used only once. The experiment was run until at least 30 battles (one-on-one) in each combination (including all intra- and interspecific interactions) were complete.

According to the Polish regulations for field and laboratory studies, neither ethical approval nor water tenant permission was required to perform this study. The studied species are not protected by law in Poland, nor listed in Annex II of the European Habitats and Species Directive. After the experimental procedure, all individuals were deprived of life by separate immersion in 70% alcohol.

The head capsule width and body weight of the larvae were measured for comparison with their winning performance; these parameters are regarded as the primary measures of condition in insects (Hynes & Coleman, 1968; Benke, 1979). To measure the width of the head capsule, a Nikon SMZ 1,000 stereoscopic microscope (with an accuracy of 0.024 mm at 40× magnification) was used. The individual weight (wet mass) of the larva was measured using a Sartorius R 160 P laboratory scale with an accuracy of 0.1 mg. SEM images (200–250× magnification) were taken of the larvae, and the length and width of the mandibles, *i.e.*, the biting part of the mouthparts, and the distance between them (Fig. 4) were measured. All SEM images were prepared in the Laboratory of Microscopic Imaging and Specialized Biological Techniques, Faculty of Biology and Environmental Protection, University of Lodz.

**Figure 4:**
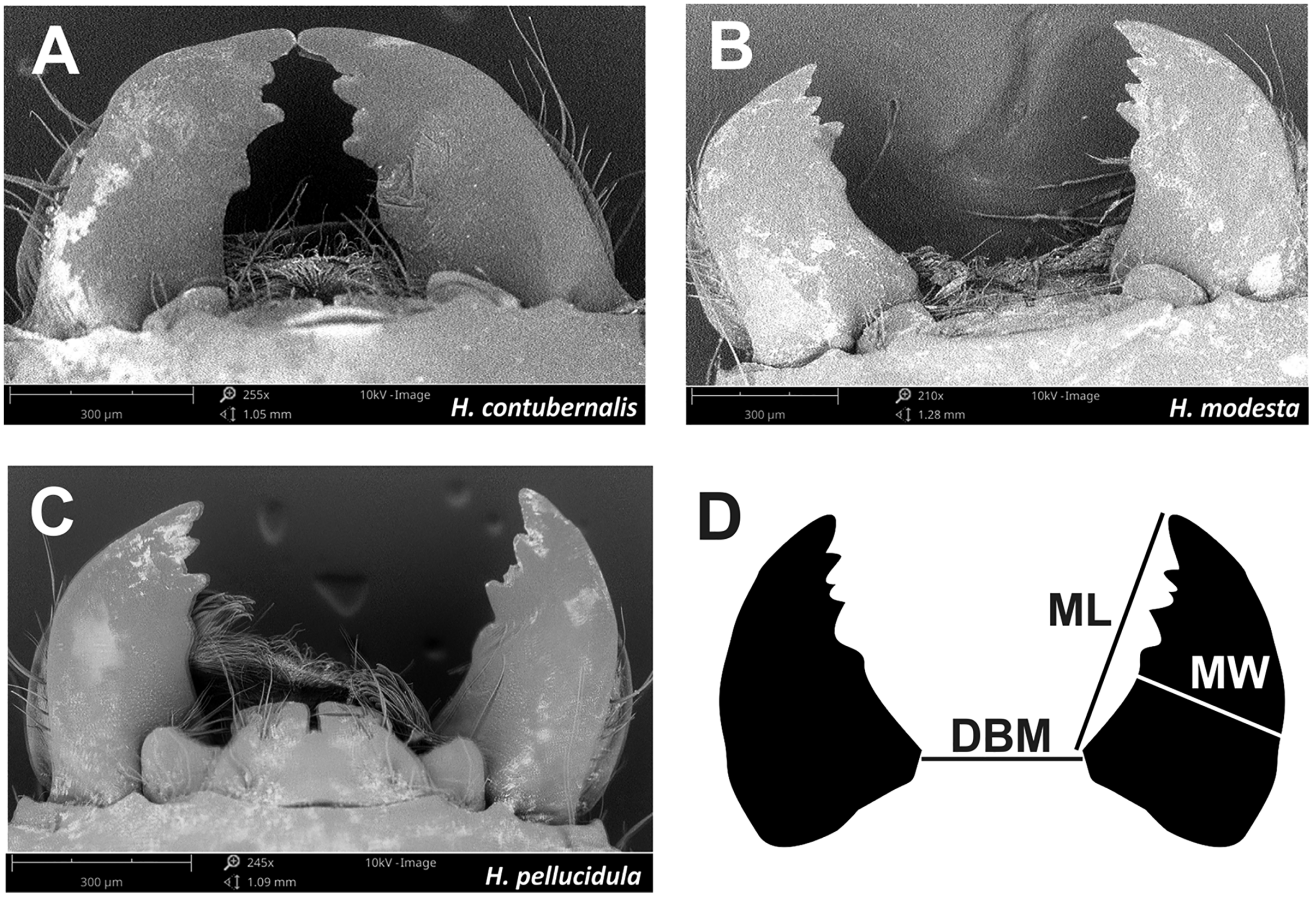
Mandibles, which are part of the chewing mouthpart used in the fight by tested species (A–C). Mandibles parameters (D) ML, mandible lenght; MW, mandible width; DBM, distance between mandibles; used for comparisons among individuals.

Data analysis was performed using the STATISTICA package (Statsoft Inc. Statistica, 2011). All continuous data sets were subjected to Kołmogorow-Smirnow test with Lilliefors correction to determine normality, and Levene’s test to confirm homogeneity of variance. All the data were log transformed (log_10_ (x + 1)) (Elliott, 1977). ANOVA and Tukey’s *post hoc* test were performed to compare the head capsule width, body weight and mandible parameters. Pearson’s r correlation was used to determine the degree of association between the studied traits of caddisfly larvae: in the case where an above-average relationship between them was observed (value_xy_ ≥ 0.5), the number of analyzed variables should be reduced. The chi-squared (χ^2^) test was used to evaluate the relationship between the tested features in a given individual and its success in combat. In all used tests, statistical significance was defined as a *p*-value of less than 0.05 (*p* < 0.05).

## Results

The conducted battles included 386 individual caddisfly larvae from the genus *Hydropsyche*: 125 specimens of *H. modesta*, 135 of *H. pellucidula*, and 126 of *H. contubernalis*. In total, 193 battles were conducted, 121 of which ended with a decisive outcome and 72 with a draw (Table 1). In case of interspecific combats, more than a half ended with a draw, while 80% of intraspecific interactions ended with a winner (Table 1). In interspecific interactions, *H. pellucidula* larvae won 21 times, *H. contubernalis* 11 times, and *H. modesta* nine times.

**Table 1:** Results of the competition between *Hydropsyche* sp. larvae used in the experiment.

Interspecific encounter larvae 1 *vs*. larvae 2	Victory of larvae 1	Victory of larvae 2	Draw
*H. contubernalis vs. H. modesta*	4	6	20
*H. contubernalis vs. H. pellucidula*	7	8	17
*H. pellucidula vs. H. modesta*	13	3	15
**Intraspecific encounter**	**One of the larvae wins**		**Draw**
*H. contubernalis vs. H. contubernalis*	24		8
*H. modesta vs. H. modesta*	28		4
*H. pellucidula vs. H. pellucidula*	28		8

Significant differences in head capsule width and individual weight were found between the studied species (F = 168.045, *df* = 2, *p* < 0.001; F = 24.218, *df* = 2, *p* < 0.001; Table 2). The highest weight and largest head capsules were observed in *H. pellucidula*, and the lowest weights and the smallest head capsules in *H. contubernalis*. The highest linear correlation coefficient for head capsule width and larval weight, treated as a physical condition factor, was observed for *H. modesta* (r = 0.511, *p* = 0.044), and the lowest for *H. pellucidula* (r = 0.313, *p* = 0.039). As these parameters did not demonstrate a high mutual relationship for all species, both features were considered for further analysis using the chi-squared test.

**Table 2:** Mean values (with standard deviation) of head capsule width (HCW) and body weight (BW) of *Hydropsyche* larvae used in the experiment including the results of combats among them.

Spec.		*H. contubernalis*	*H. modesta*	*H. pellucidula*	ANOVA
HCW (mm)		1.2780_(55)_ (0.060)	1.4137_(27)_ (0.127)	1.5560_(54)_ (0.064)	F_*(2; 133)*_ = 168.045 *p* = ***0.000***
Score	Winners	1.309_(13)_ (0.054)	1.463_(4)_ (0.105)	1.582_(18)_ (0.049)	
	Losers	1.263_(15)_ (0.063)	1.429_(7)_ (0.156)	1.518_(13)_ (0.077)	
	Draw	1.271_(27)_ (0.059)	1.395_(16)_ (0.121)	1.557_(23)_ (0.058)	
ANOVA		F_*(2;52)*_ = 2.391 *p = 0.101*	F_*(2; 24)*_ = 0.512 *p = 0.605*	F_*(2; 51)*_ = 4.244 *p* = ***0.020***	
BW (mg)		19.9_(55)_ (6.4)	25.9_(27)_ (12.9)	35.7_(54)_ (15.2)	F_*(2; 133)*_ = 24.218 *p* = ***0.000***
Score	Winners	19.0_(13)_ (0.6)	35.0_(4)_ (2.8)	41.0_(18)_ (2.0)	
	Losers	19.0_(15)_ (0.7)	35.0_(7)_ (1.8)	32.0_(13)_ (1.5)	
	Draw	21.0_(27)_ (0.6)	19.0_(16)_ (1.7)	34.0_(23)_ (1.0)	
ANOVA		F_*(2; 52)*_ = 0.603 *p = 0.551*	F_*(2; 24)*_ = 6.812 *p* = ***0.004***	F_*(2; 51)*_ = 1.474 *p = 0.237*	

**Notes:**

(_N_)–number of individuals measured for a given variant.

*(df; N-df)*–parameters of one-way ANOVA test, where df, degrees of freedom.

The p-values in bold and italics indicate statistical significances.

Regarding the 121 battles with a decisive outcome: 69 were won by the larva with wider head capsule and higher weight, while 31 were won by the larva with smaller head capsule and lower weight. In addition, the wider head capsule alone was significant in 14 cases, and the weight of the larva alone in four (Table 3). Regardless of species, larger head capsule and higher body weight were significant for winning specimens (χ^2^ = 15.059, *df* = 2, *p* < 0.001; χ^2^ = 6.914, *df* = 2, *p* = 0.008; Table 3).

**Table 3:** The relationship between size of the head capsule width (HCW), body weight (BW) and larval victory in the fight.

HCW	BW	Wins
Larger	Higher	69
Smaller	Less	31
Larger	Less	14
Smaller	Higher	4
Equal	Higher	3
Score–HCW χ^2^ = 15.059 *p* = ***0.000***	Score–BW χ^2^ = 6.914 *p* = ***0.008***	

**Notes:**

The p-values in bold and italics indicate statistical significances.

However, the winner was not always characterized by the widest head capsule and highest weight. This was most apparent in the case of *H. contubernalis* larvae, where neither parameter appeared to influence the combat result (F = 2.391, *df* = 2, *p* = 0.101; F = 0.603, *df* = 2, *p* = 0.551; Table 2). For *H. pellucidula*, head capsule width played the most significant role, while for *H. modesta* weight was more important (F = 6.812, *df* = 2, *p* = 0.004; Table 2). Only head capsule size was found to be significantly related to success among the winning larvae (χ^2^ = 17.239, *df* = 5, *p* = 0.028; Table 4); however, none of the species was favored over the others (χ^2^ = 4.879, *df* = 4, *p* = 0.300; Table 4).

**Table 4:** The relationship between size of the head capsule width (HCW) and body weight (BW) of tested *Hydropsyche* species, which ended with the victory. The number of combats was included in the brackets.

Species	Larger HCW	Higher BW
*H. contubernalis*	16 (35)	13 (35)
*H. modesta*	28 (37)	28 (37)
*H. pellucidula*	44 (49)	41 (49)
Species–score χ^2^ = 4.879 *p = 0.300*	Species–HCW χ^2^ = 17.239 *p* = ***0.028***	Species–BW χ^2^ = 9.371 *p = 0.053*

**Note:**

The p-values in bold and italics indicate statistical significances.

The analysis of the mouthparts revealed significant differences between species with regard to the length, width and distance of mandibles (F = 76.934, *df* = 2, *p* < 0.001; F = 56.765, *df* = 2, *p* < 0.001; F = 48.215, *df* = 2, *p* < 0.001; Table 5). The longest mandibles were in *H. contubernalis*, the widest in *H. pellucidula*, and the largest distance between mandibles was observed in *H. modesta* (Table 5). Out of 121 combats with a decisive outcome, only 14 were won by a larva demonstrating all three mentioned features larger than the opponent.

**Table 5:** Mean values (with standard deviation) of mandible length (ML), mandible width (MW) and distance between mandibles (DBM) in all tested larva of *Hydropsyche* including the result of the combats.

species		*H. contubernalis*.	*H. modesta*	*H. pellucidula*.	ANOVA
ML (µm)		569.3_(55)_ (24.2)	486.7_(27)_ (34.2)	563.7_(54)_ (24.9)	F_*(2; 133)*_ = 76.934 *p* = ***0.000***
Score	Winners	601.04_(13)_ (29.47)	504.87_(4)_ (32.13)	574.65_(18)_ (24.55)	
	Losers	562.08_(15)_ (29.27)	484.55_(7)_ (30.66)	555.29_(13)_ (18.43)	
	Draw	577.08_(27)_ (28.70)	484.10_(16)_ (28.19)	572.01_(23)_ (18.85)	
ANOVA		F_*(2; 52)*_ = 12.093 *p* = ***0.000***	F_*(2; 24)*_= 2.009 *p = 0.144*	F_*(2; 51)*_ = 7.434 *p* = ***0.001***	
MW (µm)		276.2_(55)_ (18.3)	316.5_(27)_ (17.8)	318.5_(54)_ (16.1)	F_*(2;133)*_ = 56.765 *p* = ***0.000***
Score	Winners	284.37_(13)_ (17.39)	317.50_(4)_ (18,82)	322.22_(18)_ (12,72)	
	Losers	268.33_(15)_ (14.58)	311.61_(7)_ (18.65)	320.67_(13)_ (15.79)	
	Draw	279.86_(27)_ (15.44)	309.77_(16)_ (21.00)	322.01_(23)_ (13.99)	
ANOVA		F_*(2; 52)*_ = 8.060 *p* = ***0.000***	F_*(2; 24)*_ = 0.565 *p = 0.572*	F_*(2; 51)*_ = 0.105 *p = 0.901*	
DBM (µm)		349.2_(55)_ (28.9)	418.5_(27)_ (25.5)	399.2_(54)_ (26.8)	F_*(2; 133)*_ = 48.215 *p* = ***0.000***
Score	Winners	362.50_(13)_ (31.49)	423.12_(4)_ (32.55)	402.78_(18)_ (25.90)	
	Losers	347.92_(15)_ (22.92)	413.39_(7)_ (31.95)	398.08_(13)_ (17.92)	
	Draw	363.19_(27)_ (29.98)	413.28_(16)_ (33.40)	400.27_(23)_ (28.20)	
ANOVA		F_*(2; 52)*_ = 2.508 *p = 0.086*	F_*(2; 27)*_ = 0.365 *p = 0.696*	F_*(2; 51)*_ = 0.266 *p = 0.767*	

**Notes:**

_(N)_–number of individuals measured for a given variant.

*(df; N-df)*–parameters of one-way ANOVA test, where df, degrees of freedom.

The p-values in bold and italics indicate statistical significances.

Irrespective of the species, longer and wider mandibles were significantly associated with a higher chance of winning (χ^2^ = 28.125, *df* = 2, *p* < 0.001; χ^2^ = 10.140, *df* = 2, *p* = 0.006); however, no such relationship was observed for the distance between mandibles (χ^2^ = 1.059, *df* = 2, *p* = 0.589; Table 6). In contrast, for all species, all three mandible parameters played significant roles in supporting the winning larva (χ^2^ = 39.613, *df* = 4, *p* < 0.001; χ^2^ = 34.092, *df* = 4, *p* < 0.001; χ^2^ = 31.443, *df* = 4, *p* < 0.001; Table 7); however, in the case of *H. contubernalis* and *H. pellucidula*, the result was determined primarily by differences in mandible length and/or width (F = 12.093, *df* = 2, *p* < 0.001; F = 7.434, *df* = 2, *p* = 0.001; F = 8.060, *df* = 2, *p* < 0.001; Table 5): the sizes of the particular parts of the mandibles did not significantly favor any of the studied species (χ^2^ = 2.288, *df* = 2, *p* = 0.318, Table 7).

**Table 6:** The relationship among size of mandible length (ML), mandible width (MW), distance between mandibles (DBM) and larval victory in the fight.

ML	MW	DBM	Wins
Larger	Smaller	Smaller	34
Smaller	Larger	Larger	24
Larger	Larger	Smaller	20
Larger	Larger	Larger	14
Larger	Equal	Smaller	10
Larger	Equal	Larger	5
Equal	Larger	Larger	5
Larger	Equal	Equal	2
Larger	Smaller	Equal	1
Equal	Larger	Smaller	1
Equal	Equal	Equal	1
Equal	Equal	Smaller	1
Smaller	Equal	Larger	1
Smaller	Smaller	Larger	1
Smaller	Smaller	Smaller	1
Score–ML χ^2^ = 28.125 *p* = ***0.000***	Score–MW χ^2^ = 10.140 *p* = ***0.006***	Score–DBM χ^2^ = 1.059 *p = 0.589*	

**Note:**

The p-values in bold and italics indicate statistical significances.

**Table 7:** Relationship among size of mandibles of tested *Hydropsyche* species, expressed as mandible length (ML), mandible width (MW) and distance between mandibles (DBM), which ended with the victory. The number of combats was included in the brackets.

Species	Larger ML	Larger MW	Larger DBM
*H. contubernalis*	35 (35)	13 (35)	2 (35)
*H. modesta*	7 (37)	30 (37)	33 (37)
*H. pellucidula*	29 (49)	30 (49)	29 (49)
Species–score χ^2^ = 2.288 *p = 0.318*	Species–ML χ^2^ = 39.612 *p* = ***0.000***	Species–MW χ^2^ = 34.092 *p* = ***0.000***	Species–DBM χ^2^ = 31.443 *p* = ***0.000***

**Note:**

The p-values in bold and italics indicate statistical significances.

## Discussion

The coexistence of closely-related species within the genus *Hydropsyche* seems to be a result of force competition related to combat (Gatley, 1988; Hemphill, 1988). The choice between competing for space and migrating to seek other suitable locations is usually dictated by the optimization of profits and losses associated with combat (Fretwell & Lucas, 1970). Larvae may demonstrate various ways of competing with each other (Englund & Olsson, 1990); however, when competing for space, there are two main reasons that lead to conflict between conspecifics or closely-related species: fighting to control new area (free space) and fighting to control an already occupied habitat. In the former case, generally the youngest larval stages (I–III) of *Hydropsyche* spp. will be fighting, with the most developed larvae competing for a suitable place for pupation (Fig. 1) (Fuller & Mackay, 1980; Coutant, 1982; Statzner, Mérigoux & Leichtfried, 2005). In the latter case, *i.e.*, already inhabited space, most research to date has focused on encounters aiming at seizing the refuge and hunting net; it was found that interactions between the intruding and resident larvae were generally aimed at the latter (Jansson & Vuoristo, 1979; Englund & Olsson, 1990; Funakoshi, 2005).

Our experimental design differed from those used in previous studies (*e.g.*, Jansson & Vuoristo, 1979; Englund & Olsson, 1990; Funakoshi, 2005), the study only included larvae without any retreat or net that they needed to defend. Such situations usually occur at the end of the larval stage, when larvae of co-existing species seek suitable places for pupation. When there is no resource to defend, there are no intruders or residents, the result of interaction should depend purely on the morphological features of the specimens: body weight, head capsule size and mandibles (Jansson & Vuoristo, 1979; Hemphill, 1988; Englund & Olsson, 1990).

Interestingly, none of the tested species was an unequivocal winner, and none of the studied features alone decided the result of the battles. Even so, in the case of interspecific rivalry, *H. pellucidula* larvae were the most successful, especially in combat with *H. modesta*, which turned out to be the most frequent loser. Victory was correlated with a large head capsule for *H. pellucidula*, and larger body size for *H. modesta*. Size and weight may represent extra potential energy resources available for combat, which might be significant in longer fights (Songvorawit, Butcher & Chaisuekul, 2018; Ebot-Ojong, Jurado & Davis, 2019); however, in the case of *Hydropsyche* spp., direct interactions are usually short, and it is more likely that body weight could offer an advantage in pushing the rival (Okada et al., 2012; Goyens, Dirckx & Aerts, 2015).

Despite this, most studies indicate that head capsule size plays a more important role in the case of Hydropsychid larvae (Jansson & Vuoristo, 1979; Englund & Olsson, 1990). Therefore, we conducted a more specific analysis of the head capsule of each specimen, including the mouthparts: these are equipped with serrated mandibles that can be used as a weapon (Englund & Olsson, 1990). Longer mandibles could increase the range of the attack (Judge & Bonanno, 2008; Chen, Hsu & Lin, 2020), while wider mandibles, with a larger area of muscle fiber attachment (Judge & Bonanno, 2008), could exert greater bite pressure, while the distance between mandibles could cause severe injury (Chen, Hsu & Lin, 2020). These serrated mandibles are the only tool which the *Hydropsyche* larvae can use in combat; they do not possess any additional attributes such as spines, outgrowths or shields, and their legs are too short to reach the rival. Therefore, it is most important that they directly face their opponent during combat (Funakoshi, 2005). Regarding the combat results, the smallest species, *H. contubernalis*, possessed the longest mandibles, which probably increased the range of the attack; *H. modesta*, with a wider head capsule, also carried wider mandibles, which were also most widely distanced. Although the caddisflies were observed to use their mandibles as weapons during their interactions, no lacerated wounds or disembodied legs were recorded. It is likely that the mandibles do not play such an important role in combat among caddisfly larvae as in ritual fights of field crickets for females (Judge & Bonanno, 2008).

Fights between the larvae of the *Hydropsyche* usually have a decisive outcome (Jansson & Vuoristo, 1979; Hemphill, 1988; Englund & Olsson, 1990); however, in our study, over a half of the performed battles ended with a draw. The fight more often ended with a decisive outcome in case of intraspecific battles (80%) than interspecific ones (44%). Intraspecific competition is usually more intensive due to the similar requirements for resources (Gatley, 1988). It is also possible that in the case of interspecific battles, the participants could more easily identify size differences with their counterparts and choose to escape the fight. Englund & Olsson (1990) report that specimens of equal size competed longer and more intensely; this might be a result of frequent intraspecific interactions, as larvae at the same stage of development of the same species are the closest in size, and it could be difficult to assess the relative fighting ability of the competitor.

Such reciprocal assessment can reduce the cost of an encounter: in cases where a significant size difference exists, it is more possible that the smaller competitor would withdraw from the fight to avoid the risk of injury or death (Arnott & Elwood, 2009). However, visual and chemical cues are probably not used when locating and assessing an opponent present under or between stones (Englund & Olsson, 1990). In running waters, a sound signal seems to be more effective (Davies & Krebs, 2009). Hydropsychidae larvae commonly employ stridulation to defend their territory (Johnstone, 1964; Jansson & Vuoristo, 1979; Silver & Halls, 1980; Englund & Olsson, 1990), and this might also carry information about the potential of the competitor (Jansson & Vuoristo, 1979). The experimental arena in our study imitated the narrow crevices present between or under the stones in small rapids, habitats preferred as suitable for pupation. The glass tube and lack of water current could have favored the signal structure (Silver & Halls, 1980). When placed in front of other larvae in a limited space, and deprived of their net-retreat, the larvae may use stridulation to defend their position: this being a less direct competition mechanism than direct combat. Jansson & Vuoristo (1979) distinguish several types of acoustic signals produced by different *Hydropsyche* species: all were classified as nonspecific “protest sounds”, although all had specific bursts, sets and periods. Although no special auditory receptors able to receive the signal have been identified within the genus (Silver & Halls, 1980), this purpose may be served by the bristles on the head or legs, in addition to measuring the speed of the current (Kaiser, 1965).

Stridulation can also be recognized as pulses (Jansson & Vuoristo, 1979), which can be propagated by the glass tube. It could be possible that in our experiment, the larvae were signaling their territory defense *via* stridulation to threaten the opponent, avoid a direct encounter or manifest fighting abilities (Jansson & Vuoristo, 1979). Growing up together, the larvae of closely-related species inhabiting the same space can collect sound information, enabling recognition of other specimens and their condition (Davies & Krebs, 2009).

However, for stridulation to be successful, both the sender and receiver of the signal must remain motionless, even if only for a moment, as the signal is generated by rubbing the head against the foreleg femora (Jansson & Vuoristo, 1979). In our study, combat never began with a violent clash, and frequent temporary stoppages were observed, which could have served as respites for situation assessment (Leimar & Enquist, 1984), and/or stridulation. Unfortunately, however, it was not possible to conduct any such measurements in the present study.

## Conclusions

Hydropsychid larvae are common and important elements of freshwater ecosystems. Various species can closely co-exist with each other, and larvae exhibit several mechanisms to avoid direct competition; however, they probably became less efficient at the end of the larval stage when large numbers of larvae simultaneously seek suitable sites for pupation. The intraspecific interactions more frequently resulted in decisive outcomes than the interspecific interactions: the larger and better-equipped competitor usually won the combat, but no particular species appeared to have any universal advantage. However, the interactions could have been influenced by several factors that were not included in our study, such as stridulation, potential experience in previous fights or volitional features, which are difficult to investigate.

## Supplemental Information

10.7717/peerj.13576/supp-1 Supplemental Information 1 Raw data. All parameters tested: combat results, head capsule width, body weight, mandibles dimensions.

## Additional Information and Declarations

### Field Study Permissions

The following information was supplied relating to field study approvals (*i.e*., approving body and any reference numbers): According to the Polish regulations for field and laboratory studies, ethical approval was not required as well as water tenant permission. Studied species are not protected by law in Poland as well as listed in Annex II of the European Habitats and Species Directive.

### Data Availability

The following information was supplied regarding data availability: The raw measurements are available in the Supplemental File.
